# High Fat, Low Carbohydrate Diet Limit Fear and Aggression in Göttingen Minipigs

**DOI:** 10.1371/journal.pone.0093821

**Published:** 2014-04-16

**Authors:** Annika Maria Juul Haagensen, Dorte Bratbo Sørensen, Peter Sandøe, Lindsay R. Matthews, Malene Muusfeldt Birck, Johannes Josef Fels, Arne Astrup

**Affiliations:** 1 Department of Veterinary Disease Biology, Section of Experimental Animal Models, Faculty of Health and Medical Sciences, University of Copenhagen, Frederiksberg, Denmark; 2 Department of Food and Resource Economics and Department of Large Animal Sciences, University of Copenhagen, Frederiksberg, Denmark; 3 Lindsay R Matthews & Associates Research International, Scerne Di Pineto, Italy; 4 Psychology Department, The University of Auckland, Auckland, New Zealand; 5 Department of Nutrition, Exercise and Sports, Faculty of Science, University of Copenhagen, Frederiksberg, Denmark; 6 Novo Nordisk A/S, Måløv, Denmark; University of Medicine & Dentistry of NJ - New Jersey Medical School, United States of America

## Abstract

High fat, low carbohydrate diets have become popular, as short-term studies show that such diets are effective for reducing body weight, and lowering the risk of diabetes and cardiovascular disease. There is growing evidence from both humans and other animals that diet affects behaviour and intake of fat has been linked, positively and negatively, with traits such as exploration, social interaction, anxiety and fear. Animal models with high translational value can help provide relevant and important information in elucidating potential effects of high fat, low carbohydrate diets on human behaviour. Twenty four young, male Göttingen minipigs were fed either a high fat/cholesterol, low carbohydrate diet or a low fat, high carbohydrate/sucrose diet in contrast to a standard low fat, high carbohydrate minipig diet. Spontaneous behaviour was observed through video recordings of home pens and test-related behaviours were recorded during tests involving animal-human contact and reaction towards a novel object. We showed that the minipigs fed a high fat/cholesterol, low carbohydrate diet were less aggressive, showed more non-agonistic social contact and had fewer and less severe skin lesions and were less fearful of a novel object than minipigs fed low fat, high carbohydrate diets. These results found in a porcine model could have important implications for general health and wellbeing of humans and show the potential for using dietary manipulations to reduce aggression in human society.

## Introduction

There is growing evidence that health as well as behavioural traits like anxiety, fear, exploration and agonistic behaviour are influenced by dietary composition, some of which we will be investigating in the present paper. High levels of fat and sugar are regular parts of a Western diet but studies of the effects of these components on behavioural traits have so far produced somewhat inconsistent results in both humans and other animals. Like the health effects, it is important to consider the contributions of different types of fats and carbohydrates on behaviour.

### Dietary sugar and behaviour

Lore and colleagues [1] found that rats given access to supplementary sucrose were significantly less aggressive in a resident-intruder test than animals not given additional sugar. In contrast, aggression was lowered in Argentine ants when deprived of sucrose [2]. Solnikh and Hemenway reported a strong positive association between high consumption of soft drinks containing sucrose and violence in Boston adolescents [3]. Further, college students who received a sugar beverage immediately before testing behaved less aggressively compared to those given a placebo, and low blood glucose levels and poor glucose metabolism related to diabetes was associated with increased aggression [4].

### Dietary fat and behaviour

Intake of fat has also been linked with proclivity for aggression, both positively and negatively. Consumption of a diet containing high quantities of n-6 polyunsaturated fats increased aggression in mice and rats [5], and dietary trans-fatty acids (originating from the hydrogenation of an unsaturated fat during food processing) associated, for example, with a decrease in n-3 fatty acids and inflammation have been linked with irritability and aggression in humans [6]. Further, increased aggression has been associated with low concentrations of n-3 polyunsaturated fat and low plasma cholesterol in dogs [7] and a high intake of n-3 polyunsaturated fat has been associated with less hostility in humans [8]. In vervets low levels of serum total cholesterol (at baseline and after receiving a high fat/cholesterol diet) were associated with high levels of agonistic behaviour and investigative behaviour [9]. Furthermore, the fat content of maternal diets might also affect offspring behaviour as a study with Japanese macaques demonstrated that the offspring of mothers consuming a diet high in saturated fat during gestation had an increased risk of being more anxious and aggressive [10]. However, no clear effect on behaviours related to spatial cognitive ability and reactivity was found for the offspring of sows consuming diets high in either saturated fat, n-6 polyunsaturated fatty acids or n-3 polyunsaturated fatty acids [11]. The inconsistent effects of dietary fat on behaviour might, in the majority of cases, arise from variations in actions of the different types of fat consumed, blood cholesterol levels and/or variations in the specific behavioural traits investigated.

### Dietary fat and general health

The view on the adverse effects of high fat intakes has changed over the years. New evidence has indicated that a high total fat intake [12] and dietary cholesterol does not adversely affect cardiovascular risk [13]. The importance for cardiovascular risk seems to concern the type of fat consumed; the adverse cardiovascular effects of saturated fat have been found to be less than those of the highly abundant poly-unsaturated fatty acid, n-6 linoleic acid [14], as well as the adverse effects attributed to refined carbohydrates [15]. Also, high fat, low carbohydrate diets have become popular, as short term studies show that such diets are effective for reducing body weight, and lowering the risk of diabetes and cardiovascular disease. In the light of these findings one can ask whether a high cholesterol, high-saturated fat diet would also have beneficial behavioural effects.

### High fat/cholesterol diet and aggression

Kaplan and colleagues [16] found that adult monkeys fed a high saturated fat (42%)/high cholesterol, low carbohydrate (39%) diet behaved less aggressively compared to monkeys offered a diet lower in fat (30%)/cholesterol and higher in carbohydrate (48%). However, the type of fat used in the high and low fat diets differed, and this difference in type (rather than amount) of fat could possibly account for the reported differences in levels of aggression. In another study where immature monkeys were fed a 40% fat (saturated, mono- and polyunsaturated), 40% carbohydrate diet, Kaplan and colleagues found that feeding supplementary cholesterol resulted in less aggression and more affiliative behaviour [17], suggesting a positive behavioural effect of cholesterol. In a pilot study, which was part of a larger study on early stage cardiovascular disease [18], Göttingen minipigs fed a standard minipig diet or a high fat/cholesterol diet also revealed differences in agonistic behaviour; the latter showing less aggression.

### The pig as a unique model

Pigs are naturally social animals, and in the wild they live in large stable groups where social interaction is highly abundant and much active time is used for exploratory behaviour. These groups are comprised of adult females, sub-adults and juveniles. Young male pigs leave the group when they reach maturity and initially stay in small bachelor groups until they are old enough to mate. Adult boars are solitary [19], [20]. The Göttingen minipig is a small pig, which is purpose-bred as an experimental animal model for use within the biomedical field. Apart from showing a slightly higher level of activity, the spontaneous behaviour and time budgets of the Göttingen minipig resemble those of other types of pigs and group housing these minipigs allows for observations of spontaneous behaviour including social interactions [21], [22]. The Göttingen minipig presents a unique model for studying the effect of diet on physiology and behaviour. It is well defined in relation to translational research and has been established as a model in obesity and diabetes research and, hence, extensive knowledge concerning physiology, metabolism and the relationship to human equivalent systems is already established [23], [24]. Like humans, pigs have a large gyrencephalic brain, they are omnivorous and have a digestive system and functioning, which is very similar to humans [25], [26]. In addition, the good perceptual and cognitive abilities of pigs [27], [28] makes it possible to test advanced problem solving in a way that resembles testing of human behaviour.

### Focus and objective of the study

The results presented here are part of a larger study where a minipig model was used for studying the effect of dietary manipulations on behaviour and cognition. In the present study we contrasted the effect of a high fat/cholesterol, low carbohydrate diet with a low fat, high carbohydrate/sucrose diet and a standard minipig diet high in carbohydrate, low in fat and sugar. We focused on aggression and fear behaviour, which were assessed in 24 young male Göttingen minipigs. Our prime interest was in determining if a high cholesterol, high-saturated fat diet could have beneficial behavioural effects. Behavioural recordings are discussed in relation to measured blood parameters and recorded skin lesions.

## Methods

### Ethics statement

Animals were treated in accordance with the Animal Experimentation Act of Denmark, which is in accordance with the Council of Europe Convention ETS 123. The study was approved by the Danish Animal Experimentation Board under the Ministry of Food, Agriculture and Fisheries of Denmark (Permit Number: 561–1434).

### Animals and Housing

The study was performed using 24 male, Göttingen specific-pathogen-free (SPF) minipigs (Ellegaard Göttingen minipigs A/S, Denmark), 8 minipigs per diet treatment (LFHC: low fat, high carbohydrate; LFHS: low fat, high carbohydrate/sucrose; HFLC: high fat/cholesterol, low carbohydrate). The animals arrived at the age of 6 weeks and were allowed two weeks of acclimatisation before the start of the study. At age 8 weeks the experimental period began, which lasted until age 21 weeks, where the study was terminated and all minipigs were euthanised by an intravenous (i.v.) overdose of pentobarbital (Pentobarbital 200 mg/ml, Glostrup Apotek, Denmark; 150 mg/kg). The study was undertaken in two replicates with 12 minipigs in each (batch A and batch B). Each batch was housed in groups of four, in pens measuring 3 m×3 m, which allowed social contact (auditory, olfactory and physical – nose to nose) between groups housed in adjacent pens (N = 2×12 = 24, n = 2×4 = 8 animals/treatment). The groups were balanced for genetic and social relatedness such that all minipigs originated from different parents/litters. In addition, if they had been housed together before their arrival, these animals were assigned to different groups. The minipigs were individually numbered with a permanent marker on forehead, flanks and back. Every morning, all pens were cleaned and provided with fresh wood shavings, straw and hay. In addition, each pen was equipped with a heating lamp which was used for pigs aged 6–12 weeks. The animal room was provided with natural lighting from three skylights as well as an 8/16-hour light-dark cycle (lights on from 7 a.m. to 3 p.m.) and filtered air at a temperature of 22°C±3°C. All behavioural tests were performed during daylight hours. The study was conducted at the Laboratory Animal Facility, Faculty of Health and Medical Sciences, University of Copenhagen, Denmark between March and November.

### Diets

During the acclimatisation period (age 6 to 8 weeks), all minipigs were fed three times daily (7.30 a.m., 11.30 a.m., 3 p.m.) with the same diet that had been used post-weaning at the breeder facility. The diet was changed slightly by the breeder between the two batches resulting in slightly different pre-test diets of batch A, Standard minipig, Piglet diet 10 kGy, Special Diets Services, UK; and batch B, Standard minipig diet, special quality control, Special Diets Services, UK (Table 1). From the age of eight weeks, the animals received the assigned experimental (pelleted cereal-based) diets three times daily throughout the study period (Table 1). The quantity of feed was calculated and adjusted weekly during the study period to meet the needs of normal growth according to age, based on the following equation: Metabolisable energy  = 1744 KJ [≈416 kcal] × Body weight^0.52^ [29]. The feed was offered in the home pen, where it was equally-distributed between four bowls. Animals assigned to the low fat, high carbohydrate diet (LFHC), served as control animals and were fed the standard minipig diet (Standard minipig diet, Special Diets Services, UK), those assigned to the low fat, high carbohydrate/sucrose diet (LFHS) received the standard minipig diet with additional sucrose (Catalogue no. 84100, purity ≥99.0%, crystal sugar, Sigma-Aldrich, Denmark A/S), and those assigned to the high fat/cholesterol, low carbohydrate diet (HFLC) were fed a modified standard minipig diet (Standard minipig diet 17% lard/2% cholesterol, Special Diets Services, UK). The sucrose was mixed with the standard minipig diet ensuring equal distribution between the four feeding bowls. In order to ensure a similar exposure to all the nutrients, apart from an increased exposure to fat and energy, the minipigs assigned to the HFLC group were offered 120 g for every 100 g fed to the minipigs in the LFHC group. The difference between these two treatments in energy intake per day (kcal) was determined, and this amount of energy was fed as sucrose to the LFHS group, hence, also adjusted weekly (79 g–115 g sucrose/pig/day). Water was provided *ad libitum* to all three groups. During behavioural tests in which a positive reinforcement (see below) was used, the minipigs received a food reward matching their respective diets i.e., LFHC animals were offered pellets of the standard minipig diet, the LFHS treatment was offered small pieces (0.5 g) of sucrose, and the HFLC treatment was rewarded with small pieces (0.5 g) of lard (Grever, OK Snacks A/S, Denmark).

**Table 1:** Pre-test diets and experimental diets.

**Pre-test diet A (age 6-7 weeks)**	**LFHC/LFHS/HFLC**		
Crude protein (kcal)	14.20%		
Crude fibre (kcal)	12.70%		
Crude fat (kcal)	4.50%		
Starch (kcal)	22.50%		
Sugar (kcal)	9.20%		
**Pre-test diet B (age 6-7 weeks)**			
Crude protein (kcal)	13.00%		
Crude fibre (kcal)	14.50%		
Crude fat (kcal)	2.10%		
Starch (kcal)	27.10%		
Sugar (kcal)	5.50%		
**Experimental diets (age 8-21weeks)**	**LFHC**	**LFHS + sucrose**	**HFLC**
Crude protein (kcal)	13.03%	10.86%	10.76%
Crude fibre (kcal)	14.52%	12.10%	11.20%
Crude fat (kcal)	2.13%	1.77%	17.51%
Starch (kcal)	27.12%	22.60%	21.83%
Sugar (kcal)	5.54%	21.54%	4.90%
**AFE (experimental diets)**			
Crude protein (kcal)	18.60%	14.34%	12.00%
Crude fat (kcal)	6.80%	5.25%	42.00%
Carbohydrate (kcal)	74.60%	80.40%	46.00%
**ME (experimental diets)**			
Total (kcal/kg)	2622.53	2861.37	2861.37

Pre-test diet A was fed to the first batch of minipigs (A) and pre-test diet B was fed to the second batch of minipigs (B) during the two weeks of acclimatisation before the test diets were applied. Low fat, high carbohydrate (LFHC); low fat, high carbohydrate/sucrose (LFHS); high fat/cholesterol, low carbohydrate (HFLC); Atwater fuel energy (AFE); Metabolisable energy (ME). Data have previously been published [47].

### Behaviour recordings and Tests

#### Spontaneous behavioural observations

The behaviour of the animals in each treatment in the home pens was video recorded weekly. One camera was placed above each pen and 7 h of video was collected once a week (9 a.m.–2 p.m., 4 p.m.–6 p.m.). The behaviours were analysed using the categories and definitions shown in Table 2 and used to calculate time budgets (using activity/inactivity behaviours) and frequencies of aggressive and other behaviours (using initiative aggressive, non-agonistic social contact; social interactions that did not involve aggression or reproductive behaviour, mounting, rooting, eating and drinking behaviours). Time budgets for each treatment group were derived from 15 min instantaneous scan samples of all animals in a pen. The frequency of aggressive and behaviours other than activity/inactivity (Table 2) was scored by focal sampling all animals in a pen, recording the occurrence of the behavioural elements by one-zero sampling every 15 min during successive 1 min periods for the 7 h weekly recordings.

**Table 2:** Ethogram of behavioural elements for the spontaneous behavioural observations.

Behavioural elements	Definition
Active	Standing upright, moving around or sitting/lying moving head around
Inactive (social)	Lying without any activity (except twitching) – in physical contact with pen mate(s)
Inactive (solitary)	Lying without any activity (except twitching) – no physical contact with pen mate(s)
Drinking	Snout in contact with water (water in bowl)
Eating	Snout in contact with feed (feed in bowl)
Rooting	Manipulating bedding, straw or hay with snout firmly to the floor
Mounting	Placing forelimbs on back of a pen mate
Non-agonistic social contact	Snout contact to any part of a pen mate's head, ears, body, tail or legs (massage-like movements might occur)
Aggression	
Biting	A rapid clear bite to any part of the pen mate's head, ears, body, tail or legs
Head knock	A rapid thrust with the head against any part of a pen mate's head, ears or body
Body pressing	Shoulder pushed hard against a pen mate, parallel or inverse parallel position, often accompanied by head knock
Levering	Snout under the body of a pen mate, lifting it from the floor

#### Back test – tonic immobility

At the age of 7 weeks, the baseline temperament of the animals was measured in a so-called Back test/Tonic immobility test [30], [31]. In this test, the minipig was lifted and placed on its back on a V-shaped cradle, with a 0.5 kg weight (10 cm×15 cm bag containing rice) placed upon the thorax. The hind legs of the minipig were gently pulled backwards. A handler placed one hand (without pressure) on the rice bag, while holding the hind legs with the other hand. This procedure was applied until the minipig became immobile, provided this happened within the first minute, where-after the handler slowly removed her hands. Hereafter, the test continued for a maximum of 3 min or until the minipig tried to escape by moving. The number of escape attempts within the first minute of the test was recorded for each minipig as well as the duration of any immobility (elapsed time from occurrence of immobility within the first minute until the first escape attempt). For animals that did not become immobile within the first minute, the test was terminated and they were assigned a duration time of 0 s. Further, the presence/absence of vocalizing during testing was recorded.

#### Animal-human familiarity test

Once a week, an Animal-human familiarity test was performed in the home pen. A familiar human handler entered the home pen and was standing still inside with their back against the wall observing the response of the minipigs to this procedure. Then the human handler sat down and reached out to each of the minipigs one by one, while observing their individual response to this procedure. Based on the observed responses, a familiarity score (see Table 3 for behavioural definitions and scoring) was calculated modified from Tsutsumi and colleagues [32] to fit the present housing and handling of the minipigs.

**Table 3:** Definitions of procedures and responses of minipigs in the Animal-human familiarity test.

Procedure	
**1) Familiar human handler is standing still inside home pen with back against the wall**	
**2) Familiar human handler sits down and reaches out to touch each minipig**	
**Response to procedure 1**	**Familiarity score**
The minipig retreats	0
The minipig stays in place	5
The minipig move towards known human handler	10
**Response to procedure 2**	**Familiarity score**
The minipig retreats	0
The minipig accepts a light touch without moving away	5
The minipig willingly receives scratching and patting, seeking social contact with human handler	10

The total familiarity score is calculated as the sum of the scorings for the two procedures, with a minimum score of 0 and a maximum score of 20.

#### Human approach test

At 17 weeks of age a Human approach test was performed. For this test, the home pen was temporarily divided into two equal sized pens by a solid wall, which prevented visual contact between the two smaller pens. One hour prior to testing, all minipigs from the respective home pens were confined to the left side of the divided pen; testing occurred on the right side (the test pen). Each minipig, in a randomly-selected order, was admitted to the test pen for a 3 min familiarisation period before being exposed to an unfamiliar human for 10 min who stood motionless and silent inside the pen with their back against one wall. The human avoided eye contact with the test animal. Latency (s) until first physical contact initiated by the minipig towards the human was recorded, total time spent in physical contact as well as the number of contacts initiated by the minipig during the test were recorded. If the minipig immediately contacted with the human when the human entered the test area, the latency was scored as 0 s.

#### Novel object test – fear test

At age 20 weeks, minipigs were subjected to a Novel object test (Dalmau and colleagues [33]), modified to fit the present housing facility and the relative small size of the minipigs, to assess fear responses (Table 4). The test area consisted of a 1 m×2.5 m arena with a manually-operated guillotine door positioned opposite the entry/exit door used by the pigs. Immediately outside the door was a holding pen. The operator of the guillotine door stood outside the test area and was not in sight of the minipigs during testing. Inside the test area a food bowl was placed 30 cm from the wall opposite the door, containing 30 g of the respective diets for the minipigs being tested, mixed with 4 g of their respective food rewards. The length of the test area was visually divided into 4 areas by marking the floor with crossing blue lines for every 65 cm. A video camera was situated above the test area to record the behaviour of the minipigs during the tests. During a pre-exposure period, once a day for three consecutive days, individual animals were trained to enter and eat food from the bowl. After training, the minipigs were tested once a day for two consecutive days; on the first (control) day there was no novel object, and on the second day, a novel object (red 40″ Balloon fender, Dan-fender, Denmark) was presented. The object was attached to the bottom of the guillotine door and was elevated 15 cm above the food bowl when presented. The animals were tested in a random order, but alternating between treatments and ensuring that each minipig was tested at the same time (between 8.30 and 10.30 a.m.) on each testing day. During the first training session, the guillotine door was already open when the pig entered. For the remaining sessions including the control and novel object test days, this door was opened to allow access to the food bowl 30 seconds after the animals entered the test area. After a further two and a half minutes, the test was terminated and the minipig was allowed to return to the home pen.

**Table 4:** Definitions of behavioural elements and locations in the Novel object test.

Behavioural element and location	Definition
Feeding behaviour	Eating feed from the bowl
Approaching behaviour	
Time spent in head position	Percentage of time spent facing the feed bowl in relation to the total time of the test
Time spend in back position	Percentage of time spent with their back to the feed bowl in relation to the total time of the test
Time spend in side position	Percentage of time spent with their side to the feed bowl in relation to the total time of the test
Locomotive behaviour	
General activity	Number of blue lines crossed during the total time of the test
Reluctance to move	The minipig has stopped for at least 2 seconds without showing exploratory behaviour
Turning back	Quick change of the body position in the opposite direction of the feed bowl
Retreat attempt	Backing away from the feed bowl
Location in test area	
Time spend in the feeding area	Percentage of time spent close to the feed bowl (<20 cm) in relation to the total time of the test
Time spend in the exit area	Percentage of time spent close to the door (<50 cm) in relation to the total time of the test

### Heart rate monitoring and recording of skin lesions

Heart rate was monitored during the Human approach test and Novel object tests. Recordings were made every 5 s on a Polar (RS800CX) training computer, Polar Electro Oy connected to a transmitter placed on a Polar WearLink - a strap placed around the minipig just behind the fore limbs. Skin lesions on ear, head, body, leg and tail regions in four pre-defined categories (Table 5) were recorded once a week. The number of lesions and severity (Smulders and colleagues [34]) were scored.

**Table 5:** Categories and definitions of recorded skin lesions.

Score	Number of skin lesions
Category 1	0–1
Category 2	2–5
Category 3	6–10
Category 4	>10

Score	Severity of skin lesions
Category 1	No lesions
Category 2	Superficial lesions
Category 3	Deep, bleeding lesions
Category 4	Highly severe lesions, where skin is torn off

### Blood sampling and analysis

After an overnight fast, venous blood samples were collected from the cranial vena cava at age 8 (baseline), 13 (medium) and 21 weeks (just before euthanasia) for analyses of a lipid profile, glucose, fructosamine and insulin. Sampling was performed on awake minipigs except on the day of euthanasia where they were sedated with a mixture of 1 mg/kg midazolam (Midazolam Hameln 5 mg/ml, Hameln Pharmaceuticals gmbh, Germany) and 10 mg/kg ketamine (Ketamine Vet 100 mg/ml, Intervet, Denmark) intramuscularly (i.m.). Subsequently, an i.v. access was provided in all pigs and 1–2 mg/kg propofol i.v. (Rapinovet Vet 10 mg/ml, Schering-Plough Animal Health, Denmark) was given if needed prior to blood sampling. After centrifugation (5 min, 2561×g at 20–25°C) samples were stored at −80°C until further analysis. Total cholesterol (TC), triglycerides (TG), high-density lipoproteins (HDL) and low-density lipoproteins (LDL) in serum were analysed using a Horiba ABX Pentra 400 Chemistry Analyser (Horiba ABX, France). Serum analyses of glucose and fructosamine were performed with an Advia 1800 Chemistry System (Siemens, Denmark), with a Siemens glucose reagent and a fructosamine reagent from ABX Pentra (Triolab, Denmark). Plasma samples were analysed for porcine insulin using Luminescence Oxygen challenging immunoassay (LOCI). For the assay, 1 µL sample/calibrator/control was mixed with 15 µL of mixture of biotinylated mAb OXI005 and mAb HUI-018-conjugated acceptor-beads in 384-well plates at 21–22°C. After incubation for 1 h at 21–22°C, streptavidin-coated donor beads were added to each well. After a further 30 min incubation at 21–22°C, chemiluminescence was measured in an Envision plate reader (Perkin Elmer). As calibrators' porcine insulin diluted in porcine plasma were used. The lower detection limit of the assay is 3 pmol/L.

### Data analysis

GraphPad Prism 5.02 was used for statistical calculations. Differences between dietary treatments for the spontaneous behavioural data were analysed using a two-way repeated measures analysis of variance. Skin lesion, Novel object test, Back test and Human approach test data were analysed using a two-tailed non-parametric Kruskal-Wallis test followed by a Mann Whitney U test for pairwise comparisons. Differences in mean heart rates between dietary treatments was analysed using a one way analysis of variance followed by a two-tailed unpaired t-test. A non-parametric two-way permutation analysis of variance adjusting for differences between batches (calculated in R; Mime.822) and a two-tailed nonparametric Mann Whitney U test for pairwise comparisons between diet treatments was used for analyses of the Animal-human familiarity test data. Differences between dietary treatments and within diets in blood parameters measured repeatedly during the study period were analysed using a two-way repeated measures analysis of variance and a paired two-tailed t-test, respectively. For differences between dietary treatments at single measurements an unpaired two-tailed t-test was applied. Analyses of differences in body weight between dietary treatments were carried out by a one-way analysis of variance followed by an unpaired t-test for pairwise comparisons.

## Results

### Spontaneous behavioural observations and skin lesions

**Figure 1:**
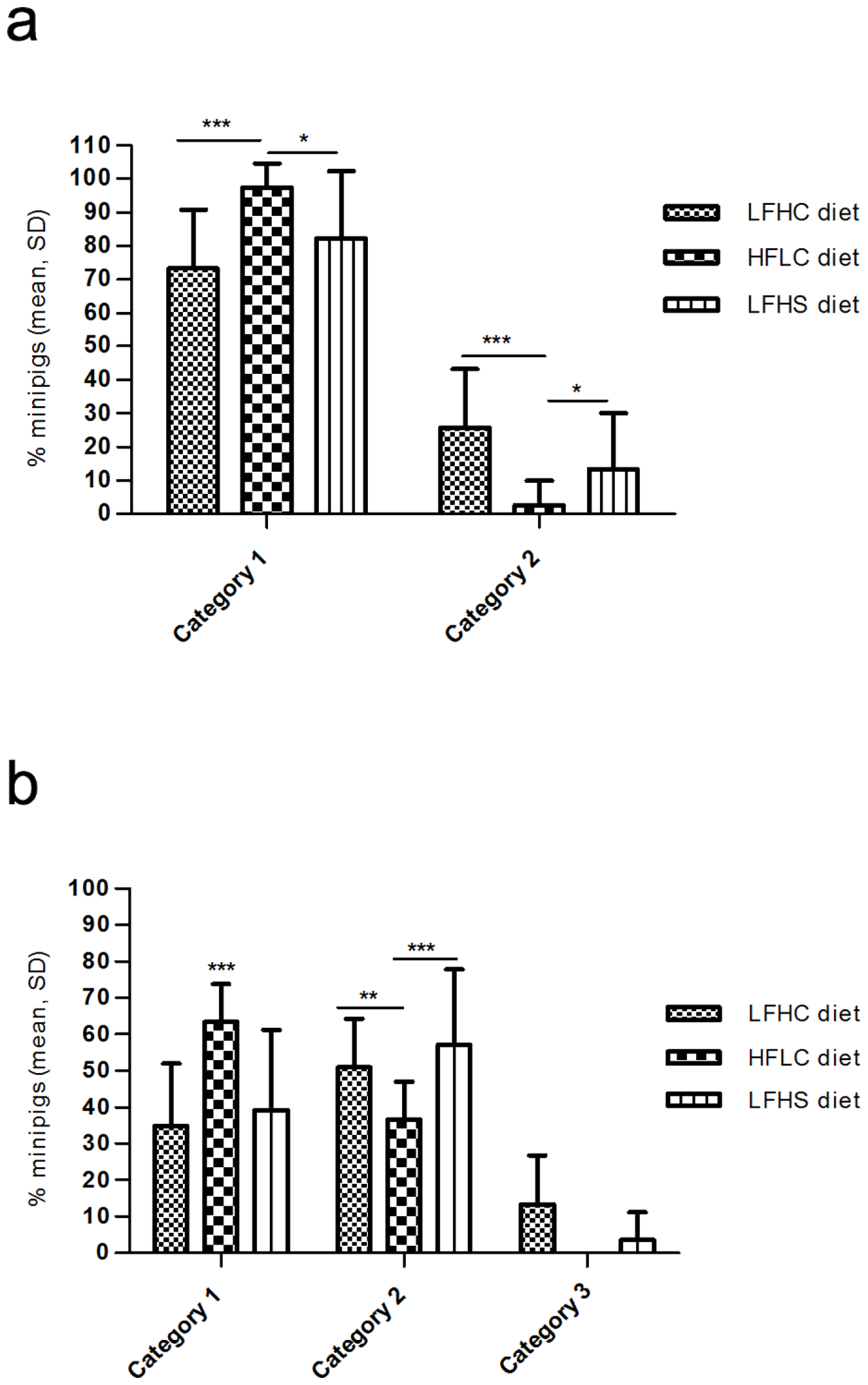
a–b. Skin lesions on head and body of minipigs. % minipigs (mean of 14 recordings ×24 minipigs and SD) with head lesions in Category 1 and 2; Category 1 (0–1 lesion), Category 2 (2–5 lesions), and body lesions in Category 1, 2 and 3; Category 1 (0–1 lesion), Category 2 (2–5 lesions), Category 3 (6–10 lesions). Low fat, high carbohydrate (LFHC); high fat/cholesterol, low carbohydrate (HFLC); low fat, high carbohydrate/sucrose (LFHS), Comparisons between the three dietary treatments were done by a two-tailed non-parametric Kruskal-Wallis test, followed by a Mann Whitney U test for pairwise comparisons. Differences between diets: *P<0.05, **P<0.01, ***P<0.001.

Table 6 shows calculations of time budget and frequencies of behaviours for minipigs in their home pen. HFLC fed minipigs showed less agonistic behaviour (F_1,168_ = 43.57, P<0.0001; F_1,168_ = 25.28, P<0.001) and more non-agonistic social contact (F_1,168_ = 17.69, P<0.001; F_1,168_ = 12.83, P<0.01) compared to minipigs on a standard minipig diet, LFHC, and minipigs fed the sucrose diet, LFHS. The proportion of time spent eating was higher for the HFLC diet compared to LFHC diet (F_1,168_ = 10.70, P<0.01). Minipigs on the HFLC diet had more category 1 (fewest lesions), and fewer category 2 lesions than animals from the other two diet treatments for both head and body lesions (Figure 1a, b). Further, although the differences were not significant, HFLC minipigs had zero severe lesions (category 3 and above, data not shown), whereas the other two treatments had instances of severe lesions.

**Table 6:** Time budget and frequencies of behaviours for minipigs in their home pen.

Behavioural elements	LFHC diet	HFLC diet	LFHS diet
Active	65%±0.08	65%±0.05	69%±0.06
Inactive (social)	25%±0.08	25%±0.04	23%±0.07
Inactive (solitary)	10%±0.04	10%±0.02	8%±0.03
Drinking	6%±0.03	6%±0.02	5%±0.02
Eating	8%±0.06	11%±0.04**LFHC	9%±0.04
Rooting	51%±0.09	44%±0.08	51%±0.07
Mounting	3%±0.02	4%±0.01	5%±0.01
Non-agonistic social contact	21%±0.03	29%±0.05**^(^*^)^	22%±0.04
Aggression	11%±0.03	3%±0.02***	10%±0.03

Data are presented as % observations (mean±SD). Low fat, high carbohydrate (LFHC); high fat/cholesterol, low carbohydrate (HFLC); low fat, high carbohydrate/sucrose (LFHS); **P<0.01, ***P<0.001. If the comparison regards only one of the other diets, this is marked by the abbreviation of the respective diet.

Results from the pilot study [18] also showed a significantly lower level (F_1,80_ = 10.47, P<0.05) of agonistic behaviour in minipigs on a high fat/cholesterol diet compared to minipigs receiving a standard diet and a non-significant trend for fewer skin lesions (Table 7).

**Table 7:** Pilot study results.

Observations	Standard diet	High fat/cholesterol diet
Aggression	7%±0.09	3%±0.05**
Body lesions (<5)	64%±0.36	82%±0.22
Body lesions (6–10)	36%±0.36	18%±0.22

Data are presented as % observations (mean±SD) of aggression and % minipigs with <5 lesions and between 6–10 lesions on the body, **P<0.01.

### Novel object test – fear test

In the Novel object test; Reluctance to move and Retreat attempts were displayed less often (P<0.05) and by fewer minipigs on the HFLC diet; 1 and 3 minipigs, respectively compared with 6 and 8 minipigs for each behavioural element on the other two diet treatments. A significantly higher mean heart rate (Table 8) was recorded in minipigs on HFLC diet compared to LFHC minipigs during the control-test in the Novel object test (P<0.05).

**Table 8:** Heart rate during Human approach test and Novel object test.

Behavioural test	LFHC diet	HFLC diet	LFHS diet
Human approach test	110±11.28	125±24.78	134±28.48
Novel object control test	122±14.64	153±24.02** **LFHC**	136±18.99
Novel object test	124±11.74	146±25.45	140±25.82

Heart rate in beats per minute presented as mean±SD. Differences in mean heart rates between dietary treatments was analysed using a one way analysis of variance followed by a two-tailed unpaired t-test,

**P<0.01 for comparison with LFHC diet; low fat, high carbohydrate (LFHC); high fat/cholesterol, low carbohydrate (HFLC); low fat, high carbohydrate/sucrose (LFHS).

### Other behavioural tests

No effects of diet were found on behaviours in Back test and Human approach test. In the Animal-human familiarity test, the familiarity score typically increased over time with no difference between diets except at age 15 weeks, where the scores decreased for LFHC minipigs (P<0.01). The familiarity score for LFHC minipigs was significantly lower than for LFHS minipigs at this time point (P<0.05).

### Blood parameters and body weight

**Figure 2:**
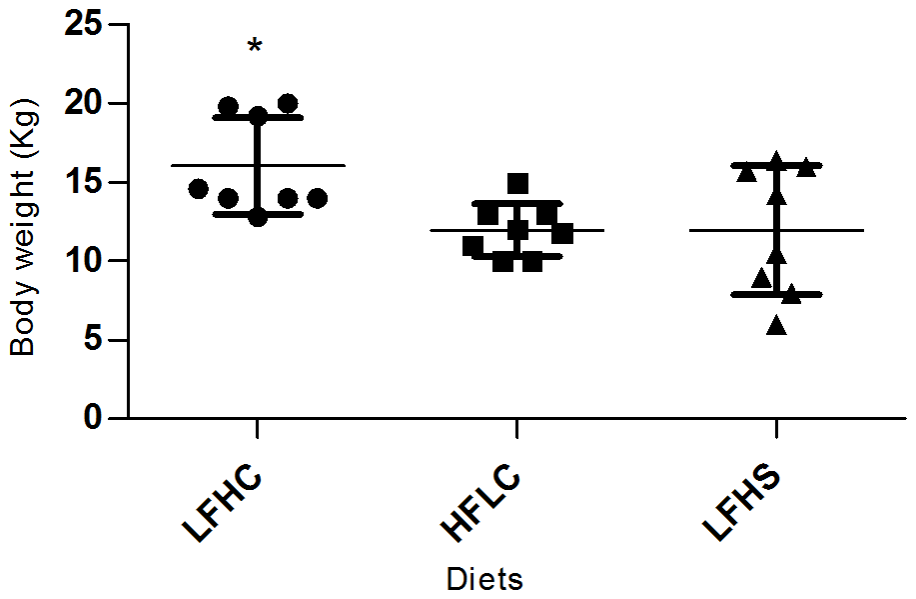
Body weight (kg) of minipigs by the end of the study. Low fat, high carbohydrate (LFHC); high fat/cholesterol, low carbohydrate (HFLC); low fat, high carbohydrate/sucrose (LFHS). Analyses of differences in body weight between dietary treatments were carried out by a one-way analysis of variance followed by an unpaired t-test for pairwise comparisons. Differences between diets: *P<0.05. Data have previously been published [47].

Results of the analyses of the blood parameters are presented in Table 9. Significantly higher concentrations of TC, LDL and HDL were found in samples obtained at age 13 weeks (medium) and 21 weeks (euthanasia), in minipigs given HFLC diet compared to minipigs on LFHC diet and LFHS diet. TC, LDL and HDL levels for HFLC minipigs were all significantly higher at the two last blood samples compared to baseline values. TG levels were significantly higher in HFLC minipigs compared to minipigs on LFHC diet at the end of the experiment. Changes in glucose and fructosamine concentrations calculated within diets revealed changes between blood sampling time points for LFHC and HFLC minipigs. At age 13 weeks, fructosamine concentrations were significantly higher in HFLC minipigs than LFHC minipigs and at the end of the experiment insulin levels for HFLC minipigs were lower than for LFHC. A difference in body weight was observed by the end of the study where the LFHC group weighed significantly more than HFLC and LFHS (Figure 2).

**Table 9:** Blood parameters.

Diets and blood samples	TC mmol/L	HDL mmol/L	LDL mmol/L	TG mmol/L	Glucose mmol/L	Fructosamine µmol/L	Insulin pmol/L
**LFHC diet**							
Baseline (B)	2.21±0.49	1.19±0.34	0.73±0.21	0.48±0.16	5.58±0.50**bE**	318.88±27.61	
Medium (M)	1.86±0.39	1.00±0.18	0.67±0.22	0.42±0.18	5.65±1.03	317.50±27.00	
Euthanasia (E)	1.39±0.22	0.88±0.13	0.41±0.10	0.33±0.07	4.80±0.41	321.50±13.85	25.03±11.88
**HFLC diet**							
Baseline (B)	2.10±0.57**bMcE**	1.07±0.24**cME**	0.78±0.40**aMbE**	0.45±0.16	5.38±0.40**aE**	321.00±18.69	
Medium (M)	16.12±9.97***	3.16±0.44***	7.44±5.90***	0.96±1.09	5.94±0.55**bE**	353.88±22.00*LFHCaBE	
Euthanasia (E)	13.18±5.71***	3.45±0.51***	5.26±2.75***	0.63±0.15***LFHC	4.84±0.41	336.38±26.71	13.61±6.86*LFHC
**LFHS diet**							
Baseline (B)	2.16±0.32	1.16±0.21	0.75±0.17	0.50±0.16	5.34±0.86	324.25±21.04	
Medium (M)	2.10±0.44	0.98±0.33	0.86±0.16	0.42±0.15	5.70±0.76	341.88±25.52	
Euthanasia (E)	1.75±0.38	1.04±0.18	0.55±0.16	0.49±0.20	4.88±0.78	331.50±18.96	19.27±14.26

Concentrations (mean ±SD) of total cholesterol (TC), high-density lipoprotein (HDL), low-density lipoproteins (LDL), triglycerides (TG), glucose, fructosamine and insulin from blood samples at baseline (B), age 13 weeks, medium (M) and at euthanasia (E). Low fat, high carbohydrate (LFHC); high fat/cholesterol, low carbohydrate (HFLC); low fat, high carbohydrate/sucrose (LFHS). Differences between diets: *P<0.05, ***P<0.001, comparisons regarding only one of the other diets, are marked by the abbreviation of the respective diet. Differences within diets: a = P<0.05, b = P<0.01, c = P<0.001, comparisons are marked by the abbreviation(s) of blood samples. TG data has previously been published [47].

## Discussion

We have shown that young male Göttingen minipigs fed a diet high in saturated fat/cholesterol and low in carbohydrate (HFLC) show less agonistic behaviour and more non-agonistic social contact compared to animals fed either a low fat, high carbohydrate (LFHC) or low fat, high carbohydrate/sucrose diet (LFHS). Moreover, fewer skin lesions were present on minipigs fed the HFLC diet, indicating a lower level of aggression in this group. A clear effect of the HFLC diet on plasma lipid levels, including higher levels of cholesterol, was evident.

Our findings support the results of Kaplan and colleagues [17] on immature monkeys, where animals receiving supplementary cholesterol behaved less aggressively. However, we cannot rule out the possibility that the effect seen in our study might be due to the combined effect of the high saturated fat together with a low carbohydrate level rather than high fat alone.

HFLC minipigs had higher heart rates during the first part of the Novel object test (control test), but on presentation of the novel object, no effect on heart rate was seen. This could indicate that these minipigs were more stressed [35], [36] or fearful during the control test, but this is not supported by the behaviour of the minipigs, since no fear-related behaviour was observed. Moreover, when presented with the novel object, HFLC minipigs demonstrated less fear-related behaviour than the two other groups. Hence, it could be suggested that these minipigs habituated more quickly to fearful stimuli or were simply less fearful, which supports behavioural findings from another study, in which, neonatal pigs had been fed cholesterol and selected for high levels of plasma cholesterol over 8 generations [37].

An underlying mechanism which might be responsible for the dietary influence on behavioural traits could be related to the serotonergic system. An association between high intake of dietary cholesterol (0.80 mg cholesterol/kcal) and higher central serotonergic activity was found by Kaplan and colleagues in their study on immature monkeys [17], suggesting a possible link between serotonin and aggression - a connection, which has later been supported by a study in dogs [38]. The HFLC diet used in our study contained 2% cholesterol, which is equivalent to ≈4.55 mg cholesterol/kcal, an amount that would most likely have an effect on the serotonergic system. A possible underlying mechanism is that the lipid composition in the brain can be altered by dietary fat intake, which in turn may contribute to possible behavioural changes [39]. Also, dietary intake of cholesterol has been found to increase the permeability of the blood-brain barrier [40] facilitating transfer of nutrients. Another possible explanation could be attributed to dietary-related changes in the composition of the gut microbiota influencing immune and inflammatory processes and the CNS, which has been shown to affect behavioural traits [41]–[43]. The underlying mechanisms could be related to the vagus nerve acting as a bi-directional communication pathway between the gut and the brain [44] and/or local immune changes in the gut causing systemic inflammation mediated by pro-inflammatory cytokines, which initiates neuroinflammation affecting the brain, leading to behavioural changes [45]. Other studies also support the idea that inflammatory pathways may be involved. Inflammatory markers like C-reactive protein have been associated with aggressive behaviour in humans especially notable in patients with Intermittent Explosive Disorder [46]. In our study no difference in the inflammatory markers C-reactive protein and haptoglobin was found between dietary treatments (data not shown here – refer to reference [47]). Presence or absence of an inflammatory state might, however, be one factor contributing to the inconsistencies reported in the literature. Diets varying in types of fat and carbohydrates content may differ in inflammatory effect - with some being anti-inflammatory others being pro-inflammatory. Our dietary treatments differed slightly in fibre content and protein content. While variation in these nutrients can influence behavioural traits [48], [49], we consider it unlikely to have had any significant effect in our study as the variations were very small. Also, the small difference in fibre content between diets was probably not relevant as all treatments were provided with additional straw and hay daily.

At the end of the study, insulin levels were lower in the HFLC group compared with the LFHC group and there was no difference in the corresponding glucose levels. A reasonable explanation for this finding is that the HFLC diet makes the minipigs more insulin sensitive as they were able to maintain glucose concentrations at lower insulin concentrations. The HFLC minipigs also had changes in blood lipids which may indicate an increased cardiovascular risk. Recent meta-analyses of randomized trials of low-carbohydrate diets with varying levels of fat (35–60%, stated in 10/23 included trials) do not suggest that these adverse changes occur in humans [50]. However, further research is needed to fully elucidate the influence of carbohydrate/fat ratio on cardiovascular risk and behavioural traits, since less aggressive behaviour might come at the expense of increased cardiovascular risk.

Increased levels of carbohydrate (in the low fat diet) in the form of extra sucrose had no effect on behaviour or the measured physiological parameters. Hence, in the minipig model used in this study, a diet high in sucrose neither increased nor decreased aggression, nor did it affect fear levels. Decreased aggression in one human study [4] was associated with a boost in blood sugar (sugar beverage) supposedly related to increased self-control as opposed to a low blood sugar level (placebo beverage) associated with increased aggression and loss of self-control. The glucose metabolism of the LFHS minipigs was not affected by the diet, which in turn would not affect behaviour, which was what we observed. Also, in light of our result, it could be hypothesised that the effects of soft drinks found in another human study [3] might have been confounded by other, uncontrolled factors in the diet and/or social factors, contributing to the association between a high consumption of soft drinks and violence.

Even though all minipigs were fed to meet growth trajectories, a minor difference between dietary treatments was found. A higher variation was seen in the LFHC and LFHS groups while there was notably less variation in the HFLC group. This could be explained by the reduced aggression observed for this group leading to less competitive behaviour during feeding time; allowing all minipigs similar access to feed and, thereby, leading to a more homogeneously fed group.

When looking at social behaviour and fearfulness of novel stimuli, our study indicates that the HFLC diet promotes less aggressive and less fearful behaviour in a minipig model, with a high translational value. Our results, hence, support a beneficial behavioural effect from consumption of a HFLC diet (with a high content of saturated fat and cholesterol). Human dietary recommendations have typically focused on reducing consumption of cholesterol and saturated fats and promoting consumption of vegetables, fruit and whole-grain foods as well as low-fat dairy products and meat in the effort to reduce cardiovascular risk. Based on the findings presented here, studies should be carried out to assess the effect of high fat/cholesterol on the social and agonistic behaviours in humans. Also, keeping in mind that associations have been made between hostility and increased cardiovascular risk factors in humans [51], it would be highly interesting to further investigate if such a diet might have beneficial effects on both general health and wellbeing. Such studies should be conducted not only in adults, but also in children. If the positive behavioural effects of a high-fat diet reported in our study were also found in humans, it should invite dietary experts and researchers in the field of human nutrition to reconsider existing dietary recommendations for healthy adults and children.

These results found in a porcine model could, hence, have important implications for general health and wellbeing of humans and show the potential for using dietary manipulations to reduce aggression in human society.
